# Bronchial thermoplasty reduces airway resistance

**DOI:** 10.1186/s12931-020-1330-5

**Published:** 2020-03-30

**Authors:** David Langton, Kim Bennetts, Peter Noble, Virginia Plummer, Francis Thien

**Affiliations:** 1 Department of Thoracic Medicine, Frankston Hospital, Peninsula Health, 2 Hastings Road, Frankston, VIC 3199 Australia; 2 Faculty of Medicine, Nursing and Health Sciences, Monash University, Clayton, Victoria Australia; 3 School of Human Sciences, The University of Western Australia, Crawley, Western Australia Australia; 4 Department of Respiratory Medicine, Eastern Health, Box Hill, Victoria Australia

**Keywords:** Bronchial thermoplasty, Asthma, Airway resistance, Imaging

## Abstract

**Background:**

The mechanism for symptomatic improvement after bronchial thermoplasty (BT) is unclear, since spirometry reveals little or no change. In this study, the effects of BT on airway resistance were examined using two independent techniques.

**Methods:**

Eighteen consecutive patients, with severe asthma (57.6 ± 14.2 years) were evaluated by spirometry and plethysmography at three time points: (i) baseline, (ii) left lung treated but right lung untreated and (iii) 6 weeks after both lungs were treated with BT. At each assessment, total and specific airway resistance (Raw, sRaw) were measured. High resolution CT scans were undertaken at the first two assessments, and measurements of lobar volume, airway volume and airway resistance were made. The Asthma Control Questionnaire (ACQ) was administered at each assessment.

**Results:**

The baseline ACQ score was 3.5 ± 0.9, and improved progressively to 1.8 ± 1.2 (*p* < 0.01). At baseline, severe airflow obstruction was observed, FEV1 44.8 ± 13.7% predicted, together with gas trapping, and elevated Raw at 342 ± 173%predicted. Following BT, significant improvements in Raw and sRaw were observed, as well as a reduction in Residual Volume, increase in Vital Capacity and no change in FEV1. The change in Raw correlated with the change in ACQ (*r* = 0.56, *p* < 0.05).

CT scans demonstrated reduced airway volume at baseline, which correlated with the increased Raw determined by plethysmography (*p* = − 0.536, *p* = < 0.05). Following BT, the airway volume increased in the treated lung, and this was accompanied by a significant reduction in CT-determined local airway resistance.

**Conclusion:**

Symptomatic improvement after BT is mediated by increased airway volume and reduced airway resistance.

## Summary at a glance

Airway resistance measured using the plethysmograph progressively reduces as bronchial thermoplasty is sequentially applied. The reduction in resistance is correlated with improved asthmatic symptom scores and with increased airway luminal volume measured by CT scanning.

## Background and aim

After three randomized controlled clinical trials, and three real-world clinical registries, we now have a chorus of evidence informing us that asthmatic patients feel better following bronchial thermoplasty (BT) [1–6]. Patients experience better symptom scores, fewer exacerbations, require less oral corticosteroids and use fewer inhalations of short-acting beta agonists. Additionally, human histological studies clearly demonstrate that BT is effective at its target site, the airway smooth muscle layer. This layer is characteristically hypertrophied in asthma, and BT induces its thinning and atrophy [7, 8]. However, it is less clear how the changes in the smooth muscle layer are translated physiologically to fewer patient symptoms.

Spirometry has been our gold standard for 100 years in the assessment of airflow obstruction, yet the anticipated changes in spirometry following BT have either been small or non-existent [4–6]. This discrepancy of symptom improvement without spirometry-based validation has been labelled a clinical paradox, and needs to be addressed to reduce physician scepticism regarding BT [9]. Other approaches have been deployed in an attempt to understand the physiological effects of BT. Lung impedance assessed by oscillometry is not affected by BT, despite improved asthma control over the same period [10]. Plethysmography has demonstrated a reduction in the degree of gas trapping (measured by residual volume) after BT, but the magnitude of this change was modest [11]. The first compelling evidence for an improvement in lung physiology after BT was presented in our recent paper, which used a CT-based segmentation methodology to demonstrate that the volume of air in the airways increased following BT treatment [12].

The aim of the present study was to determine whether the demonstrated changes in airway lumen volume after BT are sufficient to reduce airway resistance, both at the level of airways directly treated by BT, or more globally. We therefore employed two independent methods, one that used CT-based changes in lumen volume to predict local resistance, and whole lung resistance assessed by plethysmography.

## Method

### Setting and study design

This study was conducted in a university teaching hospital with a dedicated severe asthma clinic and 5 years’ experience in performing BT. Patients were referred to the clinic by their treating respiratory physician for evaluation of specialized therapies such as monoclonal antibody therapy or BT. All patients were required to meet the ERS/ATS definition of severe asthma, and needed to be using standard asthma therapy including high dose inhaled steroids and dual long acting bronchodilators, before they would be considered for further therapies [13].

In this study, the schedule of the BT procedures was altered in a novel way in order to achieve one treated lung (left side) and one untreated lung (right side). The left lower lobe was treated in the first BT session, and then the left upper lobe in the second session. Imaging assessment (CT) was performed at baseline and 4 weeks after the left upper lobe had been treated by BT, and prior to treatment of the right lung (i.e., the untreated right lung was used as a time-course control). Following the second set of imaging, the right lower and upper lobes were treated together in the final BT session. The study protocol was offered to 18 patients with severe asthma who had already chosen to undergo BT.

### Clinical measurements

The baseline data recorded included age, gender, weight, height, asthma medication usage, asthma exacerbation history, lung function parameters and the Asthma Control Questionnaire, 5-item version (ACQ) [14]. Patient assessments were performed by experienced clinical research nursing and scientific staff, and were conducted independently of the procedural team. Spirometry, diffusing capacity and body plethysmography were performed using the Jaegar Masterscreen Body (Carefusion, Hoechberg, Germany) and tests were conducted in the morning, after subjects had withheld bronchodilators during the previous day. The laboratory equipment was calibrated on the morning of testing, and all tests were conducted to ERS/ATS standards [15]. For body plethysmography, at least three acceptable measurements were performed with functional residual capacity (FRC) values within 5% of each other. The Global Lung Initiative predicted value equations were used for spirometry, whilst the ECCS predicted equations were used for plethysmography [16, 17]. Patient assessments were conducted at baseline, mid–treatment with the left lung treated and right lung untreated, and then at 6 weeks after all treatments were completed.

### Imaging studies

Non-contrast CT Scanning was performed on a 128-slice Siemens Definition AS+ scanner with a helical slice thickness of 0.6 mm, rotation time of 0.6 s, detector coverage of 38.4 mm, and tube voltage of 100 kV, consistent with the previously published technique for Functional Respiratory Imaging [18]. Two breath-hold scans were performed on each occasion – one at full inspiration (TLC), and the other at Functional Residual Capacity (FRC). Immediately prior to the CT scan, the patient was coached in the manoeuvres required for the breath-hold, and a member of the research team was present during the scan, observing the patient from the control room and providing instruction. All imaging was performed in a stable state, pre-bronchodilator, and prior to the administration of oral steroids for the BT procedure. The average estimated radiation exposure for each CT scanning session (comprising 2 scans) was 4.6 mSv, or 9.2 mSv radiation exposure for the whole study.

Post acquisition, CT images were analysed independently to the investigating team by FLUIDDA (Kontich, Belgium) The high-resolution images were imported into Mimics, a commercial, medical imaging processing software package (Materialise, Leuven, Belgium), which converted the CT images into patient-specific, 3D computer models of the lung lobes and the airway dimensions. Subsequent mathematical modelling was then performed by FLUIDDA, using proprietary Functional Respiratory Imaging (FRI) technology. FRI combines low dose HRCT images with computer-based flow simulations to quantify regional lung structure and function i.e., computational fluid dynamics.

The following parameters were calculated for each lung lobe: (i) lobar volume (ii) airway volume and (iii) airway resistance. Airway resistance was calculated from delta pressure divided by delta flow. Pressures and velocities through the airway tree were determined by numerically solving the Navier Stokes equations. The approach has been validated using SPECT CT scans, and by correlating changes in FRI-based resistance with changes in FEV_1_ after administration of a bronchodilator in asthma and COPD [18–21].

### Statistical analysis

SPSS version 25 (IBM corporation, New York, USA) was used for all statistical analyses. Grouped data is reported as mean ± standard deviation. A paired t- test was used to compare the results for post BT with pre BT, or if there were multiple sets of repeated measures, analysis of variance (ANOVA) was used. Statistical significance was taken at *p* < 0.05 for a two-tailed test.

### Ethics

The Peninsula Health Human Research Ethics Committee prospectively approved this study. All patients were enrolled having given informed consent. Women of reproductive age who were not using highly effective methods of contraception were excluded from participation so as to avoid radiation exposure to an unborn child.

## Results

### Participants

Eighteen consecutive patients, aged 57.6 ± 14.2 years (11 female, 7 male), with severe asthma participated in this study. This was a group of patients with a high symptom burden, ACQ 3.5 ± 0.9, using daily reliever medication, average 13.2 ± 9.7 puffs/day, despite very substantive preventive medication usage including oral corticosteroids in 15/18 patients. The mean oral prednisolone maintenance dose was 14.3 ± 15.8 mg/day, and the BMI was 32.1 ± 7.2 kg/m^2^. The average dose of inhaled corticosteroids was 1722 ± 826 μg/day beclomethasone equivalents, and all patients were using long acting beta-agonists. Despite this therapy, oral corticosteroid requiring exacerbations were frequent- 2.4 ± 1.8 in the 6 months prior to BT. Lung function demonstrated airflow obstruction with mean FEV1 44.8 ± 13.7% predicted, Forced Expiratory Ratio 51.1 ± 12.2%, and the bronchodilator response in FEV1 to beta-agonist was 13.3 ± 13.7%. The predominant phenotype was of Type 2-low asthma with a peripheral blood eosinophil count of 100 ± 100 cells/ul and a median IgE of 15.5(IQR 4–215). Ten of the 18 patients were never smokers. The mean pack year history was 9.7 ± 13.4 years. The diffusing capacity was 90.3 ± 28.3% predicted, and the CT emphysema score (Hounsfield units < 950) was 2.1 ± 3.9%.

### Treatment and response

**Fig. 1:**
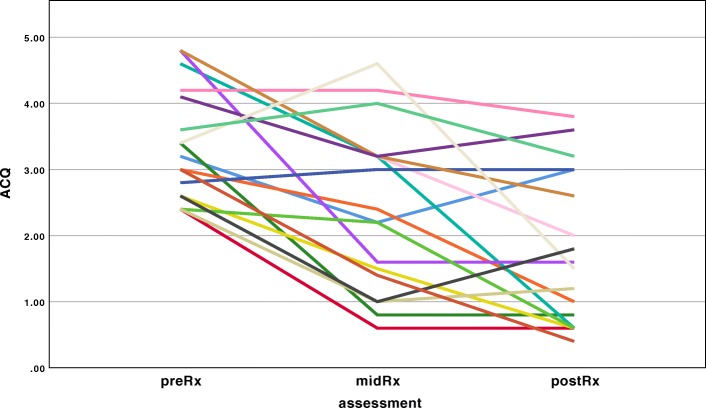
Response in ACQ to BT treatment. Footnote: Change in ACQ over time, significant at *p* < 0.001, ANOVA repeated measures

The mean number of radiofrequency activations delivered to the left lung was 110 ± 20, and the total for both lungs was 201 ± 36 activations. The ACQ score improved after sole treatment of the left lung, and improved further once the right lung was treated (Fig. 1). At the 6-week reassessment, the ACQ had fallen by 1.7 ± 1.2 units (*p* < 0.001).

### Plethysmography- volumes and resistance

The changes in spirometry and body plethysmography across the treatment period are presented in Table 1. At baseline, the plethysmograph data revealed substantive gas trapping with a Residual Volume (RV) of 154 ± 39% predicted, and Residual Volume to Total Lung Capacity (TLC ratio) of 55 ± 10%. The TLC was not elevated (103 ± 18% predicted), but the Total Airway Resistance (Raw) was markedly increased at 342 ± 173% predicted. After BT, only small improvements in spirometry were observed, 7–13%, and these bordered on statistical significance. Changes in TLC and FRC were not observed, but the RV reduced by 9% (*p* < 0.01), indicating a reduction in gas trapping, and this likely drove the increase in Vital Capacity (VC). The strongest effect in any parameter was seen in the reduction in Raw by 21% (*p* < 0.01). This was accompanied by a similar reduction in Specific Airway Resistance (sRaw) and a 34% increase in Specific Airway Conductance (sGaw).

**Table 1:** Effect of BT on Lung Function

	Before treatment	One Lung treated	Both Lungs treated	*p*
**Pre BD FEV1 (l)**	1.26 ± 0.60	1.36 ± 0.71	1.42 ± 0.68	0.069
**Post BD FEV1 (l)**	1.42 ± 0.67	1.55 ± 0.80	1.54 ± 0.71	0.052
**VC (l)**	2.46 ± 0.84	2.51 ± 0.80	2.64 ± 0.81	0.049
**TLC (l)**	5.52 ± 1.10	5.56 ± 1.19	5.51 ± 1.16	0.470
**FRC (l)**	3.48 ± 0.91	3.59 ± 1.08	3.42 ± 0.92	0.250
**RV (l)**	3.00 ± 0.70	2.94 ± 0.90	2.79 ± 0.80	0.004
**Raw (cmH**_**2**_**O.s.l**^**−1**^**)**	10.47 ± 5.30	9.96 ± 5.18	8.38 ± 5.19	0.005
**sRaw (cmH**_**2**_**O.s)**	41.1 ± 26.2	39.8 ± 26.3	33.1 ± 24.2	0.003
**sGaw (cmH**_**2**_**O.s)**^**−1**^	0.035 ± 0.024	0.039 ± 0.035	0.047 ± 0.036	0.013

*FEV1* Forced expiratory volume 1-s, *VC* Vital Capacity, *TLC* Total Lung Capacity, *FRC* Functional Residual Capacity, *RV* Residual Volume, *Raw* Total Airway Resistance, *sRaw* Specific Airway Resistance, *sGaw* Specific Airway Conductance

**Fig. 2:**
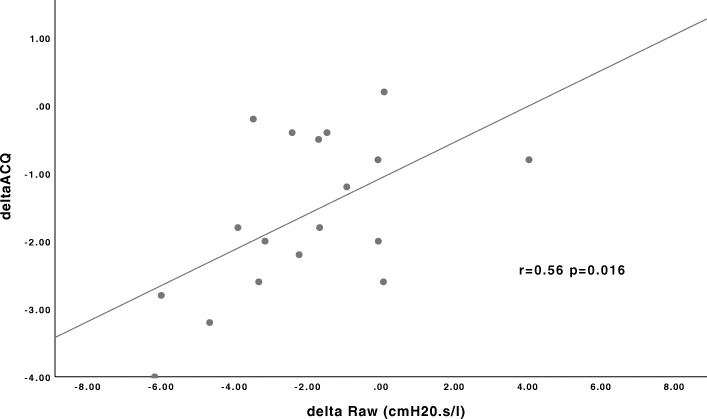
Relationship between change in Raw and change in ACQ after BT

Potential correlations between changes in lung function parameters and changes in symptom scores were examined. The strongest Pearson correlation was observed between the change in Raw and the change in ACQ, *r* = 0.56, *p* = 0.016 (Fig. 2).

### Imaging- volumes and resistance

The validity of the CT volume measurements was tested by comparison with the same measurements made in the plethysmograph. Strong agreement was observed between the two methods, (at TLC: *r* = 0.94, *p* < 0.001, at FRC: *r* = 0.90, *p* < 0.001). Changes in total lung volume and individual lobar volumes were not observed after BT.

**Fig. 3:**
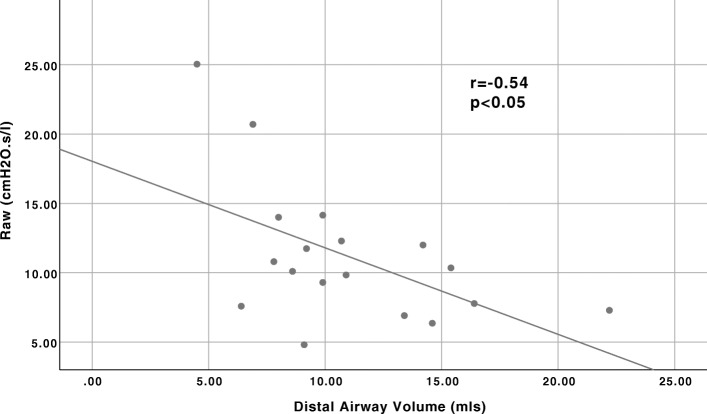
Correlation between CT derived distal airway volume and plethysmograph measured Raw at baseline

By counting CT voxels, the volume of air in the airways was determined- the limit of resolution of the technique being airways 1 mm in size. For the purposes of the analysis, the lobar and smaller bronchi were labelled distal airways, being the airways directly targeted by BT, whilst the trachea and main bronchi were labelled central airways, being untreated. The mean total airway volume at TLC was 37.2 ± 11.0 mls. This compared to an expected value of 50.4 ± 8.9 mls, based on a database of healthy, age and sex matched controls held by FLUIDDA (*p* < 0.001). The airways were segmented as described, and the volume of air in the central and distal airways was 26.2 ± 8.0mls and 11.0 ± 4.3mls respectively. The distal airway volume was 67.8 ± 22.5% predicted values (*p* < 0.001). The distal airway volume, measured by CT scanning at TLC, was inversely proportional with Raw, measured by plethysmography, *r* = − 0.536, *p* = 0.02 (Fig. 3).

**Fig. 4:**
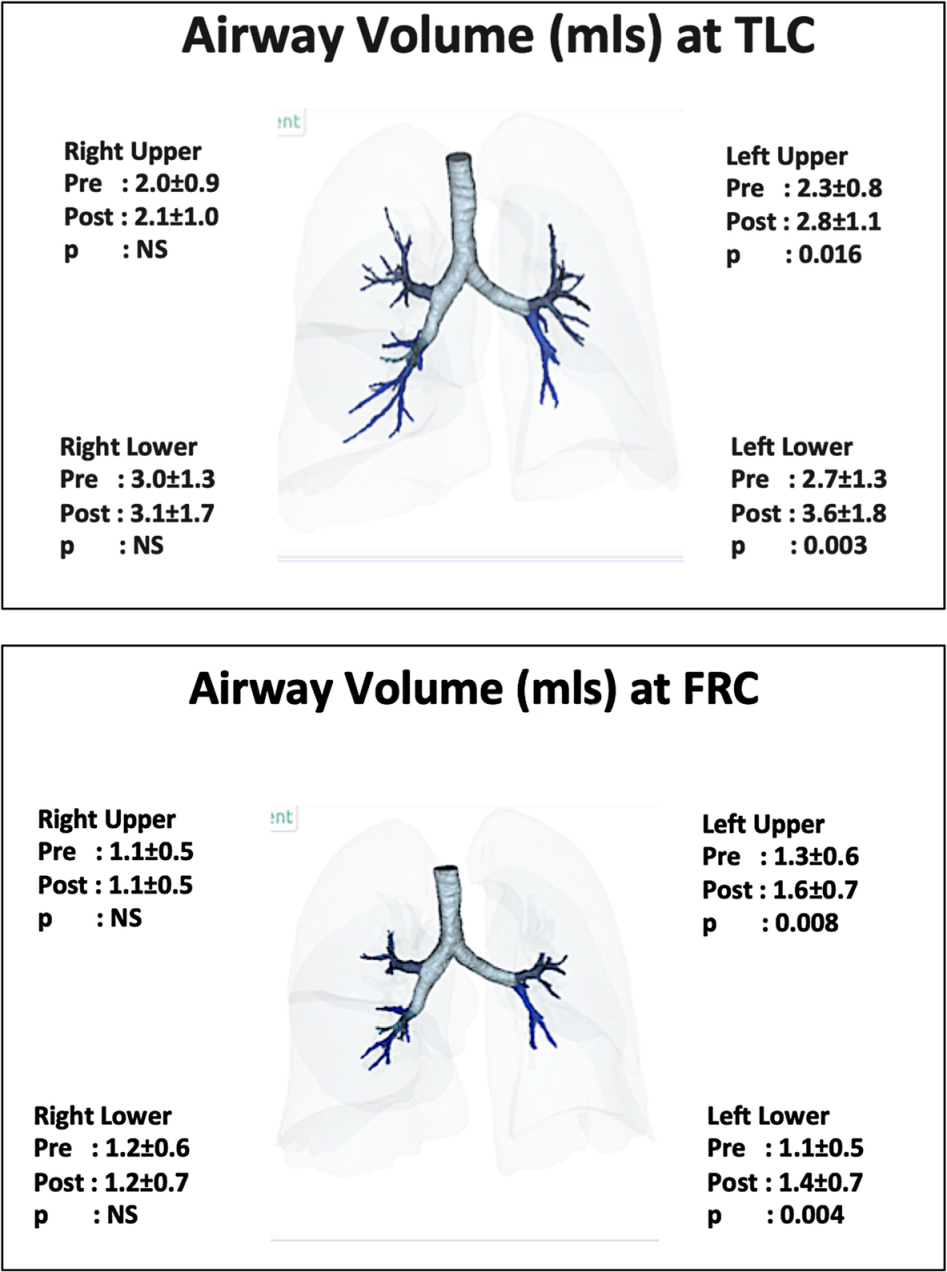
Effect of left-sided BT on airway luminal volume. Footnote: Dark blue shaded airways are potentially treated by BT, whilst light blue airways are not treated. Only left side receives treatment and right side acts as time course control

Following BT, the central airway volumes were unaffected at either TLC (preBT 26.2 ± 8.0mls, post 26.2 ± 8.8mls) or FRC (preBT 18.0 ± 6.4 mls, post 18.5 ± 6.6mls). In comparison, Fig. 4 demonstrates the changes in distal airway volume following BT. On the treated, left side, a 20–25% increase in luminal volume was identified both at TLC and FRC, and in both the left upper and left lower lobes. No changes were observed on the untreated right side in any lobe.

**Fig. 5:**
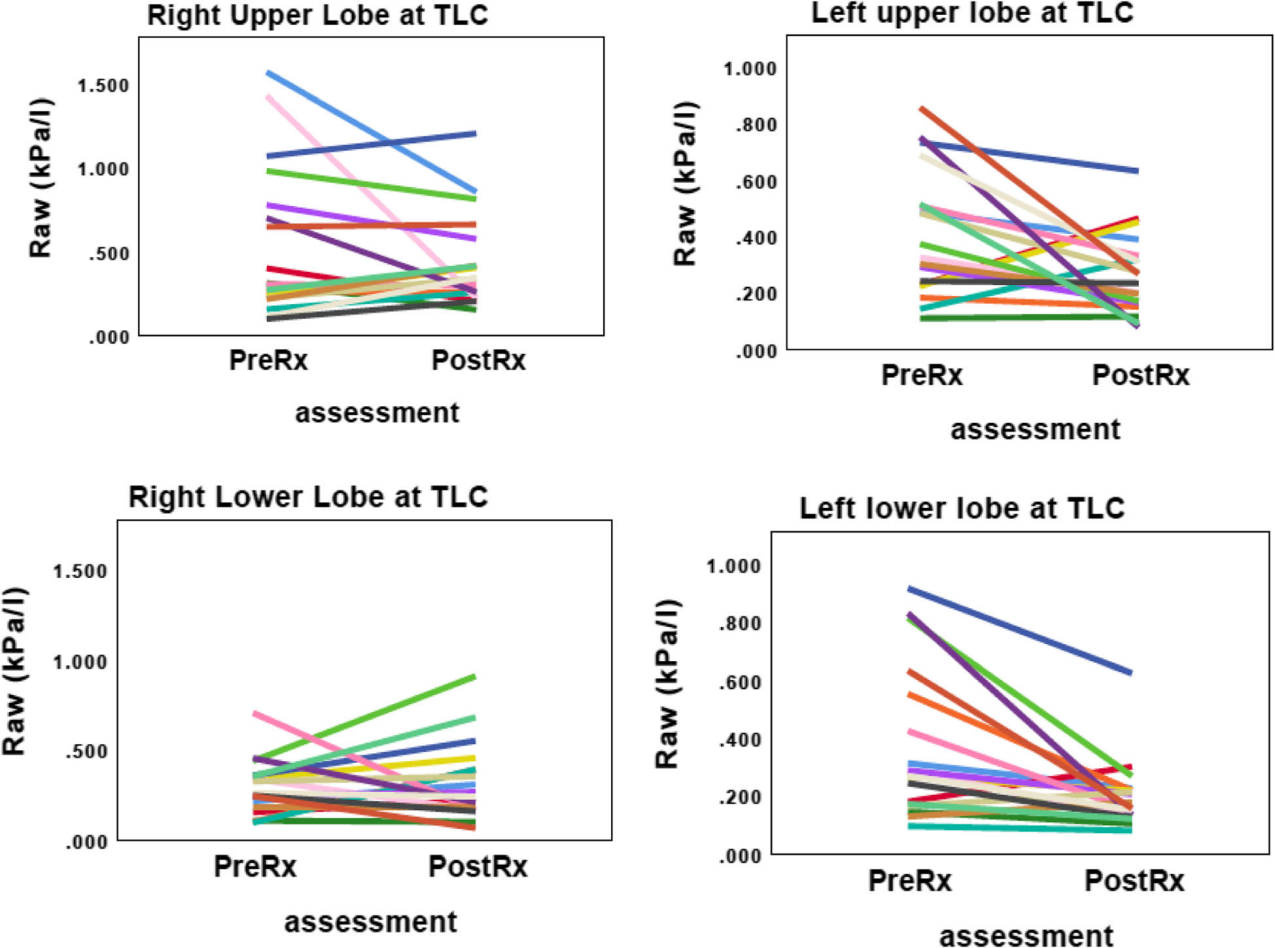
The changes in airway resistance in the upper and lower lobes measured by CT scanning. Footnote: * *p* < 0.01, pre Rx = baseline, post Rx = after left lung treated, right lung untreated

CT-derived changes in airway resistance for the upper and lower lobes of each lung before and after BT are shown in Fig. 5. Significant improvement in airway resistance in both the left upper and left lower lobes were observed following BT, whilst no changes were observed on the untreated right side. The mean airway resistance for the whole left lung at TLC improved from 0.17 ± 0.98 kPa/l to 0.12 ± 0.08 kPa/l (*p* < 0.05), representing a 29% improvement. Airway resistance in the right untreated side was 0.12 ± 0.07 kPa/l at baseline and 0.14 ± 0.08 kPa/l when reassessed (*p* = 0.40).

## Discussion

This study draws together, for the first time, the following observations after bronchial thermoplasty: (i) improvement in symptoms (ACQ), (ii) reduction in gas trapping (RV), (iii) reduction in airway resistance (Raw, sRaw) and (iv) increase in CT determined airway volume on the treated side accompanied by (v) unilateral reduction in CT determined airway resistance.

At baseline, the patients described in this study started with a high symptom burden, and very obstructed spirometry. Plethysmography revealed gas trapping and Raw was markedly elevated. CT-derived airway volumes were reduced compared with predicted values, and the reduced distal airway volumes correlated with the increased airway resistance determined by the plethysmograph. Histological studies inform us that the airway smooth muscle layer will be hypertrophied, and a positive correlation between ASM thickness and airway narrowing has been demonstrated in intraparenchymal airways from subjects with asthma [22, 23].

Following BT, histological studies consistently demonstrate atrophy of the hypertrophied smooth muscle layer, which should reduce airway wall tension and expand the airway lumen [7, 24]. Indeed, CT-derived airway volume was increased in airways targeted by BT, but constant in those left untreated. Poiseuille’s Law predicts that this should lead to a reduction in airway resistance, and this was observed to be the case in this study, using both body plethysmography and CT-based modelling. It is then intuitive, that removal of a resistant load will lead to a reduction in trapped gas at Residual Volume, which we also show. Finally, it would be expected that patients would feel better as a result of these physiological changes, and we find in this cohort of patients, a very substantive reduction in ACQ and a significant correlation between the change in airway resistance (Raw) and the change in ACQ. All of the above changes in physiology and clinical symptoms occurred in a dose dependent fashion as the treatment was progressively applied.

The observations in this study echo the early canine preclinical studies of BT, where airway calibre was assessed by CT scanning during progressive lung inflation under anaesthesia [25]. Airway size to inflation pressure response curves were measured at baseline, and then repeated with one lung treated by BT and the other an untreated control side. These studies demonstrated that BT shifted the dose response curve such that the airway calibre was greater for any level of distending pressure, thus analogous to the current human study. The effect of BT on airway resistance has also been evaluated in a mathematical model of the human lung derived from histological studies from the Prairie Provinces Fatal Asthma Study [9]. Donovan’s model predicts that with increasing ablation of the airway smooth muscle, the airway resistance falls, although this was only detectable when airway smooth muscle was in an activated state [26]. Importantly, all these approaches move beyond conventional spirometry in examining the clinical benefit of BT.

A potential limitation of the current study is that the symptomatic improvements experienced by patients after BT are large (as measured by the magnitude of improvement in ACQ), whilst the degree of improvement in Raw is modest (21%). Donovan’s model resolves this discrepancy by predicting that the downstream effects of the improved airway calibre and resistance will improve regional ventilation [9]. There is emerging evidence in studies using hyperpolarized gas magnetic resonance imaging that ventilation heterogeneity is impacted by BT [27, 28]. Thus, it seems likely that physiological effects of BT are derived from a blend of direct effects on the treated airway walls, leading to increased calibre and reduced resistance, and indirect propagated effects on the distal lung with reopening of closed airways, reduction in gas trapping and improvement in regional ventilation.

## Conclusion

In this study we demonstrate convincing physiological improvements after BT in the lungs of asthmatic patients occurring contemporaneously with reduced clinical severity.

## Data Availability

The datasets used during the current study are available from the corresponding author on reasonable request.

## Abbreviations

ACQ: Asthma Control Questionnaire (5-item version)
ANOVA: Analysis of Variance
BT: Bronchial thermoplasty
CT: Computerized Tomography
ERS/ATS: European Respiratory Society/American Thoracic Society
FEV1: 1-s Forced Expiratory Volume
FRC: Functional Residual Capacity
Raw: Total Airway Resistance
RV: Residual Volume
SABA: Short acting beta-agonists
sGaw: Specific Airway Conductance
sRaw: Specific Airway Resistance
TLC: Total Lung Capacity
VC: Vital Capacity
